# From simulation to sideline: translating finite element modeling into clinical decision-making for pediatric ACL injury and rehabilitation

**DOI:** 10.3389/fspor.2026.1868803

**Published:** 2026-06-29

**Authors:** Alexandria Mallinos, Kerwyn Jones

**Affiliations:** 1 Rebecca D. Considine Research Institute, Akron Children's Hospital, Akron, OH, United States; 2 Department of Orthopedics, Akron Children's Hospital, Akron, OH, United States

**Keywords:** anterior cruciate ligament, computational biomechanics, finite element modeling, pediatrics, subject-specific

## Abstract

Anterior cruciate ligament injuries in children and adolescents are rising, and young athletes remain at high risk for reinjury, graft failure, and early joint degeneration despite advances in reconstruction and rehabilitation. A major barrier to improving outcomes is the lack of tools that can noninvasively quantify internal knee mechanics, tissue loading, and the biomechanical consequences of surgical and rehabilitation choices in growing patients. In this narrative, we synthesized emerging work on subject-specific finite element modeling of the pediatric knee and integrated it with clinical knowledge of anterior cruciate ligament injury and management. Drawing on peer-reviewed work from multiple groups, we emphasize pediatric and adolescent applications of these modeling approaches. Rather than presenting new data, we use representative studies to illustrate how validated models have been constructed, evaluated, and applied to clinical laxity testing, ACL reconstruction variables, movement-specific ligament loading, orthotic assessment, and rehabilitation decision-making. We then highlight the striking imbalance between adult and pediatric knee modeling, emphasizing key limitations such as reliance on adult soft tissue material properties, sparse age-specific validation data, and fragmented workflows for integrating imaging, motion analysis, and simulation. Building on these insights, we propose a translational framework that links model outputs directly to clinical decision points in pediatric sports medicine, including surgical planning, brace and orthosis prescription, and return-to-sport assessment. We conclude by outlining a roadmap toward pediatric digital twin systems, in which rigorously validated, patient-specific simulations become routine tools to personalize treatment, reduce reinjury risk, and protect long-term joint health in young athletes.

## 1 Introduction

Anterior cruciate ligament (ACL) injuries are a growing concern in children and adolescents, particularly in sports that demand rapid deceleration, cutting, and pivoting. Population-based data suggests that cruciate ligament injuries in youth have increased in parallel with greater participation in organized sports and year-round competition, with adolescents who train or compete four or more times per week exhibiting markedly elevated ACL injury risk compared with less active peers (1, 2). Importantly, ACL rupture in the skeletally immature knee is not a benign event, with early cohort studies and contemporary registry data consistently showing that a childhood or adolescent ACL injury substantially accelerates the trajectory toward knee osteoarthritis and can limit lifelong participation in sports and physical activity (2, 3).

Over the past two decades, pediatric ACL reconstruction has evolved from a relatively rare and controversial procedure to a routine component of sports medicine practice. Modern physeal-sparing and transphyseal techniques can restore knee stability with a low rate of clinically evident growth disturbance when performed in experienced centers (3, 4). Nevertheless, young athletes continue to experience unacceptably high rates of graft failure and second ACL injuries. In a cohort of young athletes, those who returned to knee-strenuous sports nine months after reconstruction had a roughly sevenfold higher risk of sustaining a new ACL injury than those who delayed return (5). Analyses of high-risk populations, including professional leagues and pediatric cohorts, report graft re-tear rates approaching 12% overall, comparable to those seen in specialized pediatric series (6). More recent multicenter work in adolescents found that nearly one-third of patients sustained a second ACL injury despite acceptable patient-reported outcomes, highlighting the persistent vulnerability of this group (7). These findings underscore a critical gap: current surgical, rehabilitation, and return-to-sport strategies remain largely guided by population-level evidence and external metrics of function, within limited ability to assess patient-specific internal joint mechanics or tissue loading *in vivo*.

Clinically, decision-making in pediatric ACL care relies on a combination of history, physical examination (Lachman and pivot shift tests), plain radiography, advanced imaging, and functional assessments during rehabilitation. While these images are essential, they provide only indirect insights into the complex three-dimensional loading environment of the developing knee. MRI can depict graft integrity and tunnel placement, but cannot directly quantify graft strain, physeal stress, or cartilage and meniscal loading during sports-specific maneuvers. Similarly, return-to-sport testing protocols based on strength, hop performance, and movement quality, though prognostically useful at the cohort level, do not reveal whether a reconstructed pediatric knee is experiencing excessive ligament or cartilage loading during the pivoting and cutting tasks on the field. This limitation is particularly problematic in skeletally immature athletes, where clinicians must balance the need to restore stability and allow participation with the risks of graft overload, growth-plate injury, and early joint degeneration (2, 4).

Computational biomechanics offers a path to close this information gap. Musculoskeletal models and finite element (FE) simulations provide a means to estimate internal joint contact forces, ligament strains, and tissue stress distributions that cannot be measured directly *in vivo*. Comprehensive reviews of tibiofemoral joint FE models and ACL reconstruction simulations demonstrate that validated models can reproduce key features of knee kinematics, ligament loading, and contact mechanics under controlled boundary conditions, and can be used to systematically explore the effects of graft tension, tunnel placement, fixation strategies, and alignment on joint mechanics (8–11). At the same time, best-practice papers in computational biomechanics emphasize that meaningful translational use of FE models requires rigorous verification, validation, and transparent reporting of model structure, material properties, and simulation protocols (12–14). In parallel, broader reviews of computational modeling in joint degeneration frame FE analysis as one component of a multiscale digital twin paradigm, where patient-specific anatomy, loading, tissue adaptation, and even biological processes are integrated to support individualized risk prediction and treatment planning (15).

Despite rapid progress in adult knee modeling, subject-specific FE models for children and adolescents have only recently begun to emerge. Independent pediatric modeling work has demonstrated that atlas-based, subject-specific FE models can reproduce tibiofemoral and patellofemoral kinematics in typically developing children using MRI-based validation data (16). Subsequent neuromusculoskeletal-FE studies have extended this approach to pediatric ACL reconstruction, showing that graft type, tunnel location, graft size, and pre-tension can influence postoperative knee kinematics and cartilage stresses in a subject-specific manner (17, 18). In parallel, adolescent FE studies from our group have examined clinical laxity testing, femoral tunnel orientation, sport-specific loading, functional bracing, and ACL-deficient mechanics (19–23). Considered together, these studies illustrate an emerging but still limited pediatric FE evidence base rather than a mature clinical modeling literature.

Several limitations remain across this field. Many pediatric knee models still rely on adult-derived or literature-based soft tissue material properties, which may not capture age-dependent changes in ligament, cartilage, meniscus, or growth-plate mechanics (10, 11, 16, 24). Validation is often restricted to a narrow set of loading conditions, such as gait or quasi-static laxity tests, and only rarely extends to dynamic, multiplanar tasks such as cutting, landing, and pivoting. Moreover, clinical workflows for integrating imaging, motion analysis, and FE modeling remain fragmented, limiting real-time or near-real-time use at the point of care. These gaps motivate the present narrative.

The goal of this narrative is to chart a translational roadmap of simulation to sideline for FE modeling in pediatric ACL injury and rehabilitation. Specifically, we will (i) synthesize current evidence on patient-specific FE modeling of the pediatric knee, (ii) highlight how these models can inform surgical planning, orthotic prescription, and return-to-sports decision making in adolescent athletes, and (iii) critically evaluate methodological limitations including uncertainty in material properties, boundary conditions, and validated data that must be addressed before FE models can function as robust digital twins for the growing knee. Drawing on representative studies from multiple research groups, including selected author-led case examples, we aim to propose practical guidelines for interpreting pediatric FE outputs within sports-medicine workflows, while clearly distinguishing among evidence that is clinically actionable, evidence that supports preclinical hypothesis testing, and evidence that remains conceptual.

## 2 Methods: narrative literature identification and synthesis

This article is a narrative, hypothesis-generating perspective rather than a systematic review, scoping review, or semi-systematic review. Its purpose is to synthesize clinically relevant developments in subject-specific finite element FE modeling of the knee, with emphasis on pediatric anterior cruciate ligament ACL injury, reconstruction, rehabilitation, and return-to-sport decision-making. No new human participants, imaging datasets, simulations, or quantitative analyses were generated for this article. Previously published studies from the authors are discussed only as case examples within the broader literature.

Because this manuscript was designed as a narrative, the literature search was targeted rather than exhaustive. The review was not conducted according to PRISMA or PRISMA-ScR methodology, and we did not generate a PRISMA flow diagram, perform formal record screening, calculate inter-rater agreement, conduct risk-of-bias scoring, or produce quantitative counts of records identified, excluded, and included. This approach was selected because the aim was to provide a clinically oriented synthesis and translational roadmap rather than a comprehensive evidence map. The distinction is important because PRISMA 2020 is intended for systematic reviews, whereas PRISMA-ScR is intended for scoping reviews (25, 26). Our approach is more consistent with narrative review guidance, which emphasizes transparency of scope, balanced synthesis, and clear explanation of source selection rather than exhaustive enumeration of all studies (27).

Relevant literature was identified through targeted searches of PubMed and Google Scholar for articles published between January 1, 2000 and November 30, 2025. Search terms were used individually and in combination and included the following terms: “finite element,” “knee,” “tibiofemoral,” “adolescent,” “patellofemoral,” “anterior cruciate ligament,” “ACL,” “pediatric,” “adolescent,” “subject-specific,” “digital twin,” “graft position,” and “laxity.” We prioritized peer-reviewed studies that addressed FE modeling of knee biomechanics, ACL injury or reconstruction, pediatric or adolescent knee mechanics, subject-specific model construction, model verification and validation (V&V), and clinical translation of computational biomechanics. Reference lists of key review articles, pediatric FE studies, and methodological reporting papers were also reviewed to identify additional relevant sources.

Studies were prioritized for inclusion if they met one or more of the following criteria: they addressed FE modeling of knee biomechanics (included ACL injury, ACL reconstruction, tibiofemoral mechanics, ligament loading, cartilage contact mechanics, or pediatric/adolescent knee mechanics); developed or evaluated subject-specific or patient-specific models; reported model construction, boundary conditions, loading protocols, calibration, verification, or validation; or provided clinical context relevant to pediatric ACL injury, reconstruction, rehabilitation, return-to-sport, or growth-plate considerations. Methodological and reporting papers were included when they provided guidance relevant to FE model credibility, transparency, verification, validation, or clinical translation (12–14).

Studies were generally excluded from detailed discussion if they were animal-only studies, implant-only or bone-only FE models without relevant knee soft-tissue representation, purely multibody or musculoskeletal models without an FE component, non-peer-reviewed sources, or studies that did not address knee biomechanics, ACL injury, ACL reconstruction, model validation, or clinical translation. However, broader clinical or biomechanical studies were included when they provided necessary context for pediatric ACL injury risk, graft failure, return-to-sport concerns, or modeling assumptions.

To reduce the risk of author-study bias, author-led studies were not used as the sole evidentiary basis for any major claim. Instead, they were interpreted alongside independent adult and pediatric FE studies, broader knee FE reviews, open-source modeling resources, and FE verification/validation guidance (10, 11, 16, 24, 28–31). Author-led studies were included when they provided pediatric or adolescent examples directly relevant to laxity testing, graft orientation, sport-specific ligament loading, functional bracing, or ACL-deficient mechanics (19–23). These studies are presented as translational case examples rather than as definitive evidence of clinical utility.

The narrative synthesis was organized into four clinically relevant domains: model construction and validation; surgical planning, including graft and tunnel variables; sport- and rehabilitation-specific loading; and translational readiness for clinical or digital twin applications. Existing FE reporting and verification/validation recommendations were used as a framework for interpreting methodological strengths and limitations across studies, including transparency of geometry, segmentation, mesh construction, tissue material properties, boundary conditions, loading protocols, calibration, validation targets, uncertainty, and error reporting (12–14). The resulting synthesis was then used to develop a simulation-to-sideline roadmap linking model inputs, credibility requirements, FE-derived outputs, and pediatric ACL clinical decision points.

## 3 Narrative synthesis: evidence across the field and translational case examples

The available literature demonstrates a substantial imbalance between adult and pediatric knee FE modeling. Adult knee FE models have been widely used to examine tibiofemoral contact mechanics, ACL loading, graft positioning, meniscal injury, osteoarthritis progression, and arthroplasty-related mechanics, with several reviews highlighting both the promise and methodological challenges of these approaches (10, 11, 24). In contrast, subject-specific pediatric and adolescent knee FE models remain comparatively sparse, despite the clinical importance of ACL injury, graft failure, second injury, and early joint degeneration in young athletes (4, 32). The following synthesis therefore uses pediatric FE studies, including our own previously published work, as translational case exemplars. These examples are not presented as new results, but as illustrations of how validated modeling pipelines can be mapped to clinical decision points in pediatric ACL care. Because this synthesis was narrative rather than systematic, the studies discussed below should be interpreted as representative examples selected to illustrate clinically relevant modeling themes rather than as an exhaustive catalog of all knee FE studies.

Independent pediatric FE work has shown that subject-specific models of the developing knee can reproduce clinically meaningful joint kinematics when validated against patient-specific imaging data. Dastgerdi et al. developed atlas-based subject-specific FE models for eight typically developing pediatric participants and compared predicted tibiofemoral and patellofemoral kinematics with MRI-derived measurements from the same individuals (16). This study is important because it demonstrates that pediatric FE models can be evaluated against *in vivo*, subject-specific data rather than relying solely on adult cadaveric benchmarks. Related subject-specific knee modeling work in adults has also shown that models calibrated using noninvasive laxity measurements can produce predictions comparable to models calibrated using robotic simulator data, supporting the feasibility of living-subject model calibration (30). These studies provide a methodological foundation for pediatric digital twin development.

Author-led adolescent FE studies provide complementary case examples focused on the same clinical laxity tests used in pediatric sports medicine. In one study, 22 subject-specific adolescent tibiofemoral models were loaded to replicate standardized Lachman and pivot shift tests, and simulated anterior tibial translations were compared with historical clinical control data (21). A later ACL-deficient modeling study used similar clinical loading conditions to estimate how removal of the native ACL altered anterior tibial translation and coupled rotations, with predicted displacements compared against experimental ACL-deficient benchmarks (19, 33). These findings should be interpreted as case examples showing how FE outputs can be expressed in clinically familiar units, rather than as sufficient evidence for routine clinical use.

Surgical decision-making is one of the most developed translational targets for knee FE modeling. Adult and general ACL reconstruction FE studies have shown that graft properties, tunnel placement, fixation, and loading assumptions can influence predicted joint mechanics (11). Pediatric-specific neuromusculoskeletal-FE studies extend this concept by showing that graft type, graft size, tunnel location, and pre-tension can alter postoperative kinematics and cartilage stresses in a subject-specific manner (17, 18). Author-led adolescent FE work on femoral tunnel orientation provides an additional case example: models comparing a native ACL trajectory with a more vertical 11 o'clock femoral tunnel orientation predicted greater anterior tibial translation under Lachman and pivot shift loading (20). Together, these studies support the concept that FE modeling may help evaluate patient-specific consequences of reconstruction variables, but they also underscore the need for external validation before these simulations are used to guide surgical choices prospectively.

A smaller body of work has begun to connect external movement analysis with internal ligament and cartilage loading. In an adolescent FE study using motion-capture-derived loading, side-cutting produced substantially greater ACL and medial collateral ligament displacement than a lower-impact baseball swing within the same participants (23). These findings are consistent with broader biomechanical evidence that cutting tasks can produce high multiplanar knee moments associated with noncontact ACL injury risk (34, 35). FE modeling has also been applied to adolescent functional bracing, suggesting that patient-specific simulations may help evaluate whether orthotic designs reduce anterior tibial translation under clinical loading conditions (22). However, these applications remain early-stage and should be viewed as hypothesis-generating until replicated across larger cohorts and linked to clinical outcomes such as graft survival, second injury, or return-to-sport success.

Across the broader literature, the strongest near-term role for pediatric FE modeling is preclinical scenario testing rather than direct clinical prescription. Current models can estimate how internal knee mechanics may change across loading conditions, reconstruction strategies, bracing conditions, or rehabilitation tasks, but most pediatric models remain limited by small cohorts, adult-derived tissue properties, simplified boundary conditions, and incomplete validation across dynamic sports tasks (10, 16, 24). Therefore, pediatric FE modeling should currently be viewed as a mechanistic and hypothesis-generating tool. Movement toward clinical decision support will require stronger external validation, standardized reporting, uncertainty quantification, reproducible pipelines, prospective studies, and clinically meaningful thresholds for interpreting model outputs (12–14, 36, 37).

These pediatric FE results align with, and are reinforced by, emerging subject-specific modeling work by other groups. Andreassen et al. constructed high-fidelity adult knee FE models calibrated either to robotic knee simulator measurements or to laxity data collected with a clinical knee laxity apparatus designed for *in vivo* use (30). After calibration, the two models produced anterior-posterior laxity predictions differing by less than 2.5 mm and pivot shift kinematics within a few degrees, indicating that noninvasive clinical laxity measurements can be sufficient to calibrate subject-specific models for living knees (30). Together with open-source resources such as Open Knee, which demonstrated that shared FE models of the tibiofemoral joint can be repurposed by multiple groups to study meniscectomy, graft sizing, and ACL mechanics (38), these studies suggest that well-validated knee models can achieve accuracy levels compatible with digital-twin style applications.

Across these studies, model performance falls within or better than the ranges described in broader reviews and reporting guidelines for orthopedic FE analyses. Systematic evaluations of tibiofemoral FE models have emphasized the importance of reproducing joint laxity and contact metrics within a few millimeters and degrees of experimental benchmarks (10), and consensus documents on FE reporting and V&V stress transparent descriptions of geometry, mesh, loading conditions, and error metrics as prerequisites for clinical translation (12–14). Our pediatric models and those of others typically achieve single-digit degree agreement with experimental measures for primary kinematic outputs, while also providing ligament-level force, translation, and strain predictions that cannot be measured *in vivo* (16, 21, 30). Thus, from a translational standpoint, these modeling “results” converge on several clinically interpretable insights.

Viewed alongside clinical reviews emphasizing the absence of a clear gold standard for pediatric ACL reconstruction and the persistent risk of growth disturbance and re-rupture (4), these studies suggest that computational biomechanics may help bridge the gap between external clinical assessments and internal joint mechanics. However, the evidence should be interpreted cautiously. Current pediatric FE models can reproduce selected clinical laxity tests, explore how surgical variables may alter internal biomechanics, and estimate ligament-level loading during selected sport and rehabilitation tasks, but these outputs have not yet been prospectively validated as predictors of clinical outcomes. Accordingly, these models provide a foundation for future digital twin research rather than evidence that validated FE models are ready for routine decision-making in pediatric ACL care.

A central reason pediatric knee FE models cannot simply be scaled from adult models is that the skeletally immature knee contains anatomic structures that are both mechanically relevant and clinically vulnerable. The most important of these are the distal femoral and proximal tibial physes. These growth plates are not passive geometric boundaries; they are cartilaginous, load-bearing structures that contribute substantially to longitudinal growth and are directly at risk during ACL reconstruction. The distal femoral physis and proximal tibial physis have complex three-dimensional shapes, and their area and volume change with age before plateauing during adolescence (39). In clinical ACL reconstruction, these physes constrain tunnel location, tunnel diameter, tunnel obliquity, graft fixation, and the choice between physeal-sparing, all-epiphyseal, hybrid, partial transphyseal, and transphyseal techniques (40, 41).

For FE modeling, the physis should therefore be treated as a pediatric-specific anatomic region rather than ignored or merged into surrounding bone. When the clinical question involves ACL reconstruction, the model should ideally represent the femoral and tibial physes as separate segmented structures, quantify the distance between graft tunnels and the physes, and estimate the volume or area of physeal violation when transphyseal or partial transphyseal tunnels are simulated. These outputs are clinically meaningful because physeal damage can produce angular deformity, limb-length discrepancy, overgrowth, or altered tibial slope (42, 43). Thus, in pediatric surgical simulations, “acceptable” restoration of anterior tibial translation is not sufficient; the model must also evaluate whether the simulated graft path, fixation, and tunnel geometry respect growth-plate anatomy.

The pediatric ACL itself also changes during growth. Pediatric and adolescent patients demonstrate age-related changes in ACL diameter, length, orientation, and tibial attachment position, with the ligament becoming more vertically oriented and more posteriorly attached as development progresses (41). These developmental changes are clinically relevant because graft diameter, graft trajectory, and tunnel placement must fit within the intercondylar notch and epiphyseal bone stock without overstuffing the notch, impinging on the roof or lateral wall, or crossing vulnerable physes. For FE models, this means that the native ACL or reconstructed graft should not be represented as a generic adult ligament scaled by body size alone. Instead, ligament line of action, cross-sectional area, insertion sites, pre-strain, and bundle behavior should be matched as closely as possible to skeletal maturity and patient-specific imaging.

Osseous morphology is another important pediatric-specific feature. Intercondylar notch width, tibial eminence morphology, posterior tibial slope, coronal alignment, and rotational profile may influence ACL injury pattern, graft impingement, and internal joint loading. Pediatric ACL injuries may also present differently from adult injuries because younger children may sustain tibial spine avulsion fractures rather than midsubstance ACL rupture when the physis or osteochondral attachment fails before the ligament substance (44, 45). MRI-based pediatric studies have also associated lateral posterior tibial slope and notch morphology with ACL injury risk in children and adolescents (46, 47). These anatomic variables should be incorporated into subject-specific FE models when the intended use involves injury risk, graft loading, reconstruction strategy, or return-to-sport loading.

Finally, pediatric soft tissues cannot be assumed to behave identically to adult tissues. Cartilage, menisci, ligaments, and physes are all influenced by growth, maturation, loading history, and injury status. Yet most pediatric FE models still rely on adult-derived or literature-based tissue properties because pediatric tissue testing data are sparse. This limitation is especially important when interpreting outputs such as cartilage contact stress, meniscal load, ligament strain, graft displacement, or physeal stress. In the near term, pediatric FE models should explicitly report whether tissue material properties are pediatric-specific, subject-specific, adult-derived, or assumed. In the longer term, quantitative MRI, longitudinal imaging, and multicenter pediatric datasets may allow age- and maturation-specific material-property assignment for cartilage, meniscus, ligament, and growth-plate tissues.

## 4 Translational roadmap from simulation outputs to clinical decisions

A central challenge for pediatric ACL finite element modeling is translating technically complex simulation outputs into clinically interpretable information. To become useful at the point of care, model outputs will need to be linked to decisions that clinicians already make, including surgical timing, graft and tunnel planning, brace or orthosis prescription, rehabilitation progression, and return-to-sport counseling. Table 1 summarizes a proposed simulation-to-sideline roadmap that links patient-specific inputs, modeling steps, simulation outputs, clinical interpretation, and evidence-readiness levels. The roadmap is intended to clarify translational requirements, not to imply that current pediatric FE outputs are ready to determine patient-specific treatment decisions. For skeletally immature patients, this roadmap must also include pediatric-specific anatomic checkpoints, including skeletal maturity, physeal status, epiphyseal tunnel feasibility, graft diameter relative to notch and ACL size, tibial slope, and the relationship of reconstruction tunnels or fixation devices to the distal femoral and proximal tibial physes.

**Table 1:** Simulation-to-sideline translational roadmap for pediatric ACL finite element modeling.

Translational stage	Patient-specific inputs or modeling requirements	FE-derived outputs	Clinical decision point	Current evidence-readiness level	Key validation requirement before routine clinical use
1. Patient-specific data acquisition	Clinical history, skeletal maturity, physical examination, radiographs, MRI, motion analysis, sport participation, and injury mechanism	Patient-specific anatomy, alignment, growth-plate status, baseline laxity, and movement patterns	Risk stratification, surgical timing, rehabilitation baseline, and sport-specific counseling	Clinically available, but not yet routinely integrated into FE workflows	Standardized pediatric imaging and motion-analysis protocols that can be used without excessive cost, radiation, or clinical burden
2. Model construction	Segmentation of femur, tibia, cartilage, menisci, ligaments, grafts, physes, and epiphyseal bone stock; mesh generation; assignment of pediatric-specific or clearly justified tissue material properties; definition of tunnel-physis relationships and boundary conditions	Subject-specific knee model capable of estimating internal mechanics under controlled loading	Determines whether the model is anatomically and mechanically appropriate for the child being evaluated	Feasible in research settings; limited automation for clinical use	Reproducible segmentation, mesh convergence testing, pediatric-specific material-property data, and transparent model reporting
3. Verification and validation	Numerical verification, mesh sensitivity, comparison with laxity tests, MRI-derived kinematics, cadaveric data, or *in vivo* measurements	Model credibility metrics, such as agreement in anterior tibial translation, rotation, contact mechanics, or ligament displacement	Determines whether model outputs are credible enough for the intended clinical question	Emerging evidence in pediatric and adult subject-specific knee models	Context-of-use-specific validation, external replication, uncertainty analysis, and reporting consistent with FE and computational model credibility guidance
4. Surgical scenario simulation	Alternative graft types, graft diameters, tunnel positions, tunnel orientations, fixation assumptions, graft pre-tension, and physeal-sparing or transphyseal strategies	Anterior tibial translation, coupled rotation, graft displacement or strain, cartilage contact pressure, and potential physeal loading	Graft selection, tunnel planning, reconstruction strategy, and assessment of over-constraint or under-constraint	Preclinical scenario testing; not yet definitive for prospective surgical planning	Prospective comparison of FE-predicted mechanics with postoperative laxity, graft survival, growth disturbance, and patient-reported outcomes
5. Rehabilitation and sport-specific loading	Motion-capture-derived loads, ground reaction forces, muscle forces, task selection, cutting, landing, pivoting, running, and sport-specific drills	Ligament displacement, graft demand, cartilage stress, meniscal loading, and task-specific mechanical risk	Rehabilitation progression, exercise selection, timing of exposure to cutting or pivoting, and return-to-sport counseling	Hypothesis-generating and early preclinical evidence	Longitudinal studies linking FE-derived loading metrics to reinjury, graft failure, symptoms, and return-to-sport success
6. Brace and orthosis simulation	Brace geometry, strap placement, joint constraints, patient-specific anatomy, loading conditions, and compliance assumptions	Change in anterior tibial translation, rotational control, ligament displacement, and joint loading with and without bracing	Brace prescription, orthosis design, activity-specific bracing recommendations, and patient counseling	Early-stage proof of concept	Validation against instrumented brace studies, clinical laxity measures, patient adherence, comfort, and reinjury outcomes
7. Clinical interpretation and feedback	Integration of model outputs with physical examination, imaging, surgical plan, rehabilitation milestones, and patient goals	Decision-support summary showing expected mechanical consequences, uncertainty, and evidence-readiness level	Shared decision-making among surgeon, rehabilitation team, athlete, and family	Conceptual to early translational	Prospective multicenter testing, clinician usability studies, outcome-based thresholds, and regulatory/implementation guidance
8. Pediatric digital twin updating	Repeated imaging when clinically indicated, serial functional testing, wearable sensors, rehabilitation data, and longitudinal outcomes	Updated estimates of graft maturation, joint loading, cartilage stress, and reinjury risk over time	Longitudinal personalization of rehabilitation, sport reintegration, and monitoring after ACL reconstruction	Conceptual future direction	Automated pipelines, longitudinal validation, data governance, interpretability standards, and evidence that model-guided care improves outcomes

Importantly, this roadmap does not imply that current pediatric FE models are ready to independently determine treatment decisions. Rather, it clarifies the translational steps required for these models to move from mechanistic research tools toward clinically useful decision-support systems. Current evidence is strongest for reproducing controlled laxity tests and exploring preclinical “what-if” scenarios, such as graft orientation, ACL deficiency, sport-specific loading, and bracing effects (19–23). Evidence is also emerging for pediatric model validation and pediatric ACL reconstruction parameter testing using neuromusculoskeletal-FE workflows (16–18). However, prospective validation against clinical outcomes remains necessary before FE-derived thresholds can be used for routine clinical decision-making.

The proposed roadmap therefore separates model capability from clinical readiness. A model may be capable of estimating anterior tibial translation, ligament displacement, graft loading, cartilage contact stress, or physeal stress, but clinical use requires additional evidence that these outputs are accurate, reproducible, interpretable, and associated with patient outcomes. This distinction is consistent with FE reporting recommendations and computational model credibility frameworks, which emphasize transparent model description, verification, validation, uncertainty assessment, and context-specific credibility before model outputs are used to support clinical or regulatory decisions (12–14, 36, 37).

## 5 Discussion

The emerging pediatric FE literature suggests that subject-specific knee models may provide clinically interpretable estimates of internal joint mechanics that are difficult or impossible to measure directly *in vivo*. However, the current evidence base remains early-stage. Independent pediatric FE studies have established important validation benchmarks for typically developing knees and pediatric ACL reconstruction simulations, while adult and general subject-specific knee studies have advanced methods for calibration, validation, reproducibility, and model sharing (10, 16–18, 28–30). Author-led adolescent FE studies extend these concepts to clinical laxity testing, graft orientation, sport-specific loading, bracing, and ACL-deficient mechanics, but these studies should be interpreted as translational case examples rather than definitive evidence of clinical efficacy (19–23). The roadmap in Table 1 provides a practical structure for interpreting these applications. Rather than treating FE outputs as direct clinical instructions, the roadmap frames them as decision-support variables whose value depends on the intended clinical context, the credibility of the model, and the consequences of an incorrect decision. For example, a simulation used to compare graft tunnel orientations in a preclinical research setting may require a different level of validation than a simulation used to recommend return to pivoting sports for an individual athlete. This context-specific interpretation is consistent with computational model credibility principles, which emphasize that the rigor of verification, validation, and uncertainty assessment should match the model's intended use and decision risk (36, 37).

From a surgical perspective, pediatric FE simulations suggest that technical variables such as graft tunnel orientation, pre-tension, and graft size may influence postoperative knee kinematics and cartilage loading in growing knees (16–18). Multi-parameter NMSK-FE pipelines have shown subject-specific variation in tibiofemoral translation and cartilage stress during gait, with no single surgical configuration emerging as universally optimal across modeled knees (16–18). Similarly, adolescent FE models evaluating femoral tunnel orientation suggest that more vertical tunnel positions may increase anterior tibial translation under simulated Lachman and pivot shift loading (20). These findings support the use of FE modeling for preclinical “what-if” analyses, but they do not yet justify patient-specific surgical recommendations without prospective validation against postoperative laxity, graft survival, growth disturbance, patient-reported outcomes, and long-term joint health.

FE modeling may also help connect rehabilitation and return-to-sport assessments with estimates of internal tissue loading. Current return-to-sport decisions often rely on strength, hop performance, movement quality, time from surgery, and patient-reported outcomes, but these measures do not directly quantify graft strain, cartilage stress, or ligament displacement during cutting and pivoting tasks. Simulation-based estimates of internal loading could eventually complement these tools by identifying tasks that appear externally acceptable but generate high internal mechanical demand. At present, however, this application remains investigational. Existing pediatric and adolescent FE studies provide proof-of-concept evidence, but larger prospective studies are needed to determine whether FE-derived metrics improve reinjury prediction, rehabilitation progression, or long-term joint preservation beyond current clinical assessment methods (10, 23, 24, 32). Despite these promising examples, pediatric FE knee modeling remains dramatically underdeveloped relative to the adult literature. Contemporary reviews of knee FE analysis and ACL reconstruction modeling largely synthesize studies conducted in skeletally mature populations, spanning osteoarthritis, total knee arthroplasty, meniscal pathology, and adult ACL reconstruction (10, 11, 24, 28, 31, 48, 49). In contrast, only a small number of studies have developed and validated subject-specific FE models of the pediatric or adolescent knee, and even fewer have systematically explored ACL deficiency or reconstruction scenarios in this age group (16–23). A recent pediatric FE validation study explicitly notes that most FE knee models are primarily developed for adult knees, with pediatric populations being neglected from this area of research (16), underscoring the magnitude of this gap.

Several factors are likely to contribute to this disparity between adult and pediatric knee FE modeling. First, high-resolution imaging suitable for segmentation is more challenging to justify in children without a clinical indication, and ethical constraints limit access to pediatric cadaveric specimens for validation. Second, pediatric anatomy introduces structures and modeling requirements that are often absent from adult models, including open distal femoral and proximal tibial physes, evolving epiphyseal bone stock, age-dependent ACL size and orientation, and changing cartilage, meniscal, and ligament properties (39, 41). Third, the clinical consequences of modeling error are different in a growing knee. A tunnel orientation that appears mechanically acceptable based on anterior tibial translation may still be clinically inappropriate if it violates the physis, creates asymmetric physeal stress, over-constrains the joint, or increases cartilage contact stress (42, 43). As a result, pediatric FE models must evaluate not only knee stability, but also growth-plate safety, graft-physeal relationships, epiphyseal tunnel feasibility, and age-appropriate joint loading.

These anatomic considerations also affect validation. Adult FE models are often validated against cadaveric kinematics, arthroplasty mechanics, or osteoarthritis-related contact stresses, whereas pediatric ACL models require validation targets that are meaningful for growing athletes. These may include MRI-derived tibiofemoral and patellofemoral kinematics, clinical laxity measures, anterior tibial translation during Lachman testing, pivot-shift kinematics, graft or ligament displacement, cartilage contact stress, and physeal stress or tunnel intersection metrics (16, 19, 21). Future pediatric FE work should therefore report skeletal maturity, physeal status, growth-plate segmentation strategy, graft tunnel relationship to the physes, and whether material properties were pediatric-specific, subject-specific, or extrapolated from adult data.

### 5.1 Methodological challenges for clinical translation

Beyond the population imbalance, there are important methodological barriers that must be addressed before pediatric FE models can be fully embedded into routine decision-making. First, the assignment of patient-specific soft tissue material properties remains a major source of uncertainty, but the problem is more complex than simply selecting ligament or cartilage stiffness values. Pediatric models must account for tissues that are actively maturing, including cartilage, menisci, ligaments, subchondral bone, and the physes. Growth plate cartilage is mechanically and clinically distinct from epiphyseal and metaphyseal bone; therefore, models that merge the physis into surrounding bone cannot estimate physeal stress or tunnel-related physeal injury. Similarly, models that assign adult-derived ligament properties to pediatric graft tissue may misrepresent laxity, coupled rotation, and graft strain in a child or adolescent. These limitations should be explicitly reported, and sensitivity analyses should be used to determine how uncertainty in pediatric material properties affects clinically important outputs such as anterior tibial translation, graft displacement, cartilage stress, meniscal load, and physeal stress (10, 16, 24, 39, 41). Second, realistic boundary and loading conditions remain challenging, particularly for dynamic tasks. Many FE studies still rely on simplified compressive loads and prescribed flexion angles, which do not fully capture the muscle-driven, multi-planar loading that characterizes both sports maneuvers and clinical laxity tests (24, 48). Sequentially linked neuromusculoskeletal-FE models, such as those recently applied to pediatric ACL reconstruction, represent an important advance by integrating subject-specific kinematics and muscle forces derived from gait analysis (16–18). However, extending these pipelines to cutting, landing, and other high-risk movements will require more complex motion capture protocols, robust inverse dynamics, and efficient numerical solvers to keep computational times within clinically acceptable limits (48).

Third, rigorous V&V are non-negotiable prerequisites for clinical use. Consensus recommendations in orthopedic and trauma biomechanics emphasized transparent reporting of mesh convergence, constitutive model assumptions, numerical solver settings, and validation against experimental or *in vivo* data (12, 13). Many existing knee FE studies, particularly earlier ones, fall short of these standards, limiting reproducibility and clinician confidence. Encouragingly, recent pediatric FE work has explicitly benchmarked model predictions against arthrometric measurements, instrumented Lachman tests, and historical pivot shift data, achieving close agreement in anterior tibial translation and rotational laxity across multiple knees (16, 20, 21, 23). These studies provide a blueprint for pediatric-specific V&V, but broader adoption of standardized checklist and multi-site validation will be essential for regulatory acceptance and clinical integration.

### 5.2 Positioning pediatric FE models within the broader ACL literature

When viewed alongside classical cadaveric and adult FE studies, pediatric models both confirm and extend established principles of ACL biomechanics. Foundational adult FE work has helped delineate how anterior tibial translation, valgus moments, and internal rotation loads contribute to ligament strain and failure, and how notch geometry or tibial slope may predispose individuals to noncontact injury (9, 35, 50). Adult subject-specific FE studies have also linked altered cartilage contact pressures after ACL injury or reconstruction to regions prone to osteoarthritic degeneration, reinforcing the concept of mechanical overloading as a driver of long-term joint damage (10, 24, 48).

Pediatric FE simulations build on this foundation by demonstrating that adolescent knees do not simply behave as scaled-down adults. Differences in epiphyseal geometry, ligament orientation, and joint laxity across growth stages produces age-specific patterns of translation, rotation, and tissue stress under identical external loads (4). Simulations of ACL reconstruction in pediatric knees further suggest that parameter combinations deemed acceptable in adult practice may over-constrain or under-constrain growing joints, potentially exacerbating cartilage overloading or graft failure risk if applied without adjustment. These insights align with clinical concerns about high re-injury rates and early osteoarthritis in young ACL patients and support calls for truly age-specific surgical strategies and rehabilitation progressions (4, 32).

### 5.3 Pediatric digital twins for ACL injury and rehabilitation

The final stage of the roadmap is the development of pediatric ACL digital twins: longitudinal, patient-specific models that are updated as a child grows, heals, rehabilitates, and returns to sport. In this framework, the FE model is not a static research simulation but a living decision-support tool that incorporates new clinical information over time. Preoperative imaging and motion analysis could define the initial model. Intraoperative choices such as graft type, tunnel location, and graft tension could be simulated as alternative scenarios. Postoperative rehabilitation data could update loading assumptions and return-to-sport testing or wearable sensor data could refine estimates of task-specific ligament and cartilage loading. This vision is consistent with broader computational modeling and digital twin concepts in musculoskeletal disease, but pediatric ACL applications remain early and require careful validation before clinical deployment (15, 18, 24, 48).

For pediatric ACL care, the most realistic near-term use of digital twin-style modeling is likely selective application in complex or high-risk cases rather than routine use for all patients. Potential future applications include comparing reconstruction strategies in silico, estimating mechanical demand during rehabilitation tasks, and evaluating whether brace designs or movement modifications reduce ligament or cartilage loading. These applications should be framed as research and preclinical decision-support scenarios until prospective studies establish accuracy, clinical utility, feasibility, cost-effectiveness, and outcome benefit.

### 5.4 Limitations and future directions

This article should be interpreted as a narrative rather than a systematic review, scoping review, or semi-systematic review. Although we added a transparent description of the databases searched, search terms, date range, source-selection priorities, and general exclusion criteria, the search strategy was not designed to meet PRISMA or PRISMA-ScR standards. As a result, the literature selection remains narrative and partly expert-driven, which may introduce selection bias, limit reproducibility, and reduce the ability to assess the comprehensiveness of the review. Some relevant studies may not have been captured, particularly given the rapidly evolving literature on pediatric ACL injury, subject-specific FE modeling, neuromusculoskeletal-FE pipelines, model validation, and digital twin methods. The conclusions should therefore be interpreted as a clinically oriented synthesis and translational framework rather than as an exhaustive evidence map.

As a narrative, this manuscript is inherently selective rather than exhaustive. The FE studies highlighted here were chosen to illustrate a translational pathway from simulation to sideline, rather than to comprehensively catalog all knee modeling efforts. In particular, we focused on continuum FE models of the tibiofemoral joint, other modeling approaches such as multibody dynamics or reduced-order surrogate models, may also play important roles in real-time decision support but were not discussed in depth. Furthermore, most of the pediatric FE studies to date, including our own, are limited by small sample sizes and retrospective designs, and few have directly linked FE-predicted mechanics to long-term clinical outcomes such as graft survival or osteoarthritis progression (16, 20, 21, 23). The proposed roadmap should therefore be interpreted as a translational framework rather than a validated clinical algorithm. Each step will require prospective testing, external validation, and outcome-based thresholds before FE-derived metrics can be used to guide patient-specific treatment decisions.

A major limitation of the current pediatric FE literature is that model outputs have rarely been linked prospectively to clinical outcomes. Most available pediatric and adolescent FE studies are small-cohort, retrospective, validation-focused, or preclinical scenario analyses. Although these studies can reproduce selected clinical laxity measures, estimate tissue loading, and explore surgical or rehabilitation variables, they do not yet establish that FE-guided decisions improve graft survival, reduce second ACL injury, prevent growth disturbance, accelerate safe return to sport, or reduce long-term osteoarthritis risk. The proposed roadmap should therefore be interpreted as a translational research framework rather than a validated clinical algorithm. Prospective, multicenter studies with standardized model reporting, external validation, uncertainty quantification, and outcome-based thresholds are needed before FE-derived metrics can guide patient-specific treatment decisions (12–14, 36, 37).

A further limitation is that pediatric-specific anatomy and tissue properties remain incompletely characterized for FE modeling. Although MRI can define patient-specific bone, cartilage, meniscus, ligament, graft, and physeal geometry, most available pediatric models still rely on simplified or adult-derived material properties. This is particularly important for simulations that estimate cartilage contact stress, graft strain, meniscal loading, or physeal stress, because these outputs may be sensitive to age-dependent tissue behavior. Future studies should prioritize pediatric imaging protocols, normative anatomic datasets, quantitative MRI, physeal segmentation, and maturation-specific material-property estimation. Until these data are available, pediatric FE studies should clearly distinguish between outputs that are strongly supported by validation and outputs that remain sensitive to uncertain anatomic or material assumptions.

Despite these limitations, the convergence of pediatric FE modeling, high-quality clinical imaging, and motion analysis provides an important opportunity to study internal knee mechanics in young athletes. The central message of this narrative is not that pediatric FE models are ready for routine clinical decision-making, but that they can help define mechanistic hypotheses, identify patient-specific modeling challenges, and guide the design of future validation studies. With targeted investment in pediatric-specific data, standardized V&V practices, and closer collaboration between modelers and clinicians, FE-based digital twins of the pediatric knee may evolve from research tools into clinically useful decision-support systems.

## 6 Conclusion

Subject-specific FE modeling of the pediatric knee has advanced enough to generate clinically meaningful hypotheses and, in selected validated workflows, support preclinical scenario testing for pediatric ACL injury and rehabilitation. Existing models can reproduce clinically relevant laxity tests, evaluate how reconstruction variables may alter knee mechanics, and estimate ligament loading during sport-specific tasks that cannot be measured directly *in vivo* (16–18, 20, 21, 23). However, pediatric FE modeling remains substantially less developed than adult knee modeling literature, and most available studies remain limited by small sample sizes, incomplete pediatric material-property data, simplified loading assumptions, and limited prospective clinical validation (10, 11, 24). Therefore, the most appropriate near-term role for pediatric FE modeling is to support mechanistic understanding, preclinical testing, and individualized hypothesis generation rather than routine clinical decision-making.

Closing this gap will require pediatric models that move beyond adult-derived, homogeneous tissue properties toward age- and region-specific descriptions of cartilage, menisci, ligaments, and growth plates, driven by quantitative imaging and targeted experimental data. Loading conditions must better capture muscle-driven, multiplanar dynamics in cutting and landing tasks, and FE pipelines must be streamlined, standardized, and embedded within robust V&V frameworks to earn clinician and regulatory trust. Ultimately, we envision pediatric digital twins in which subject-specific FE models are generated from routine imaging and motion analysis and used prospectively to test surgical plans, brace designs, and return-to-sport progressions before implementation. Thus, pediatric knees cannot be treated, biomechanically or computationally, as scaled-down adults. The simulation-to-sideline roadmap proposed here provides a structured pathway for translating validated FE models into pediatric ACL care, moving from patient-specific data acquisition and model credibility assessment to preclinical scenario testing, clinical decision support, and eventually longitudinal pediatric digital twins. However, clinical adoption will require transparent reporting, external validation, uncertainty quantification, multicenter replication, prospective outcome studies, and clear evidence thresholds for each intended use.
